# High content screening for drug discovery from traditional Chinese medicine

**DOI:** 10.1186/s13020-019-0228-y

**Published:** 2019-02-28

**Authors:** Jing Wang, Ming-Yue Wu, Jie-Qiong Tan, Min Li, Jia-Hong Lu

**Affiliations:** 1 State Key Laboratory of Quality Research in Chinese Medicine, Institute of Chinese Medical Sciences, University of Macau, Macao SAR, China; 2 Key Laboratory of Medical Genetics, Xiangya Medical School, Central South University, Changsha, Hunan China; 3 Mr. and Mrs. Ko Chi Ming Centre for Parkinson’s Disease Research, School of Chinese Medicine, Hong Kong Baptist University, Hong Kong SAR, China

**Keywords:** Drug discovery, High-content screening, Advanced models, Progressive instruments, Traditional Chinese medicine

## Abstract

Traditional Chinese medicine (TCM) represents the crystallization of Chinese wisdom and civilization. It has been valued as the renewable source for the discovery of novel drugs, owing to its long-term proved efficacy in human diseases and abundant biologically active components pools. To dissect the mystery of TCM, modern technologies such as omics approaches (proteomics, genomics, metabolomics) and drug screening technologies (high through-put screening, high content screening and virtual screening) have been widely applied to either identify the drug target of TCM or identify the active component with certain bio-activity. The advent of high content screening technology has absolutely contributed to a breakthrough in compounds discovery and influenced the evolution of technology in screening field. The review introduces the concept and principle of high content screening, lists and compares the currently used HCS instruments, and summarizes the examples from ours and others research work which applied HCS in TCM-derived compounds screening. Meanwhile, this article also discusses the advantages and limitations of HSC technology in drug discovery from TCM libraries.

## Introduction

The advancement of optical instruments greatly accelerated the process of modern biology and the drug discovery industry [1]. Fluorescence microscopy emerged as a robust tool substituted for conventional optical equipment, which can analyze spatiotemporal information in biology to uncover the mysterious veils of cellular events [2]. Simultaneously, the development of molecular biology system attributes to the rapid growth of biological probes and fluorophores. After the image acquisition, thousands of figures are scanned to analyze quickly by computational software. Compared with manual screening technique, automatic screening platform avoided the assay artifacts and subjective biases on effective targets to achieve more accurate experiment results. Moreover, the automated drug screening platform saved manpower and resources, and increased the speed and scale of drug screening, which greatly accelerated the drug discovery process. In the early stage of the drug discovery, high throughput screening (HTS) system was extensively used in searching for hit compound for its high-efficiency, high-speed and quantitative characteristics. However, the single-target identification approach sometimes could not meet the need for comprehensive evaluation of compound activity in such a huge compound libraries generated by TCM or chemical synthesis [3]. HCS as a multiple dimension approach, displayed unique strength both in target-based and phenotypic-based screening for drug discovery.

### Principle of high content screening

The concept of the high content screening was first proposed in 1997, when it was regarded as a powerful approach to break the bottlenecks in drug discovery [4]. Identifying a hit compound from a large number of compounds libraries requiring the robotic instruments and automatic analysis. Characteristics of high content screening meet the request at the following aspects. First, the establishment of multiple parameters and targets analysis platforms can extract unbiased information on cellular function and morphology at the same time, such as cell shape, growth, differentiation, translocation, apoptosis and metabolism [5]. Second, investigators acquire spatial and temporal information on cellular events in vitro. In this way, researchers can mimic in vivo conditions to evaluate effective treatments on intricate diseases. Third, the robust approach provides more insights into mitochondria, lysosome and nucleus activity to study the subcellular biological events. Finally, lead compound validation by automated imaging analysis and data algorithms made HCS easier to be extensively applied. Above mentioned characteristics of HSC make it widely used by researchers all over the world for the identification the active lead compound [6].

### State-of-the-art progress in HCS technology

Hitherto, numerous high technologies and assays were established to improve the high-content imaging system in the biological field. Diversity of instruments were invented for devising complete experiments and acquiring multiple data analysis. Nowadays, multi-channel detectors have been widely used in imaging analysis systems, enabling the simultaneous analysis of multidimensional targets and phenotypes. Accordingly, several software packages have been implanted to optimize the experiment operation for screening. Meanwhile, Open-source image analysis software has been continuously developed for HCS image-analysis to acquire information in spatial and temporal dimensions [7], including both quantitative and qualitative assays [8]. These softwares aimed at analyzing specific imaging problems and providing user-friendly operation, can be extensively used in HCS equipment such as cell cognition [9], ImageJ/Fiji [10], and EBImage [11]. 3D tissue culture model is a novel technology in biology that researchers acquired tridimensional phenotypes of cells by confocal microscopes [12]. 3D culture assay is an ideal tool to explore cancers, specific organs from stem cells, circulatory and nervous system diseases between monolayer cell culture with animal experiment. Associated with a large number of confocal HCS platforms, the 3D model system attempted to act as a new approach in drug discovery pipeline. 3D culture instruments including the PerkinElmer Opera which contained a spinning disk confocal microscope, the ImageJ Suite combined with an R tool [13], and 3D Object Counter by Fabrice P. Cordelieres [14] have been applied in the drug screening on 3D culture-based models.

The application of HCS technology in biological field or pharmaceutical industry firmly bounded to the improvements of hardware, especially in microscopic imaging system and image-analysis software [15]. To a certain extent, both advanced imaging technology and data analysis software caused further development of HCS approaches. These two aspects, as a breakthrough in the exploration and improvement of HCS, make drug screening technology develop rapidly. Over the past 20 years, tremendous changes have taken place in automated microscopes. The adequate resolution and magnification are necessary to capture subcellular structures and phenotypes which reflect the cell events. With the progress of microscope technology and automated imaging system, HCS technology evolved rapidly. It become easier for researchers to get a better overview of cellular phenotypes in organisms, and to identify the hit compounds from the huge compounds library [16].

Currently, advancement of microscopic technology expanded the range of automated screening for visual phenotypes [17]. Improvements in stable light source and fast autofocus spelt the growth of microscopy techniques. In addition to this, the process in fluorescent probes and novel fluorescent proteins also contributed to expanding the visual phenotypes [18]. Fluorescent labeling assay can be employed to visualize the complicated physiological activity of cells in image-based screening. Fluorescent dyes, fluorescent probes, genetically encoded fluorescent proteins and antibodies allowed direct monitoring of cellular phenotypes under complex conditions through staining cells [19]. Changes in image-based approaches and reliable software workflow facilitate to extract multiple parameters or quantitative information from images. Both robotic microscopy imaging technique and advanced computational analysis software were the rudimentary components of HCS technology. HCS technology combined these two aspects in automated experiment process and avoided of time consuming or uncertainty by human. Under the development of experimental technology, the classification of instruments in HCS has changed with fantastic progress. Currently, HCS detector can be segregated into three categories: high-content of wide field fluorescence microscopy, high-content of confocal fluorescence microscopy, and integration of above two. The current HCS instruments have concluded as follows (Table 1).

**Table 1:** Current HCS instruments

	Company	Name	Light source	Website
Confocal microscope	GE Healthcare	InCell Analyzer 6000	Laser	http://www.biacore.com
	Lecia Microsystems	TCS SP5	Laser	http://www.leica-microsystems.com
	Molecular Devices	ImagreXpress Micro	Laser	http://www.moleculardevices.com
	Perkin Elmer	Opera	Laser	http://www.perkinelmer.com
Wide-field microscope	GE Healthcare	InCell Analyzer 2000	Halide lamp	http://www.biacore.com
	Intelligent Imaging Innovations	3i Marianas	Arc lamp	http://www.intelligent-imaging.com
	Molecular Devices	ImageXpressMICRO XLS	Arc lamp	http://www.moleculardevices.com
	Olympus	Scan^R	Arc lamp	http://www.olympus.com
	TTP LabTech	Acumen eX3	Laser	http://www.ttplabtech.com
Confocal combined with	BD biosciences	BD Pathway 855	Arc lamp	http://www.bdbiosciences.com
Wide-field	BD biosciences	BD Pathway 435	Arc lamp	http://www.bdbiosciences.com
	Perkin Elmer	Operetta	Arc lamp	http://www.perkinelmer.com
	Thermo Scientific	Cellomics ArrayScan VTI	Arc lamp	http://www.cellomics.com
	Thermo Scientific	CellInsight CX7	LED	http://www.cellomics.com
	Thermo Scientific	CellInsight™ CX5	LED	http://www.cellomics.com

### HCS applications in drug discovery

**Fig. 1:**
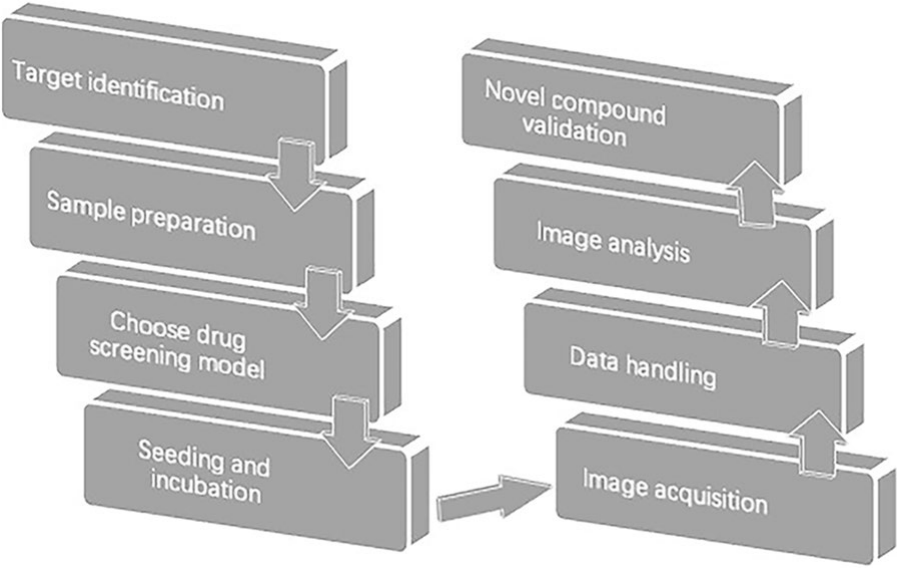
Key steps in high content screening. Upper flow chart illustrates general process of high content screening. Each step is required to precisely design and optimize

High content screening is a technology that combined automated fluorescence microscopy with automated image analysis to track the cellular morphology and intracellular parameters. As a contemporary technology, it differs from the traditional cell-based methods, because it can analyze multiple cell parameters and thousands of individual cells simultaneously (Fig. 1).

Drug target validation has been taken into account to be a pivotal procedure in drug discovery owing to know target points lead the way of discovering novel drugs. Identifying the target point facilitate to have a specific pharmacological mechanism and decide whether the drug screening will be a success. Historically, hundreds of drug targets have been determined, such as a receptor, enzyme, ion channel and nucleic acid [20]. Although the research of drug targets made an enormous progress, there are still unexploited fields need further exploring. In the past years, scientists spared their efforts to study the mechanism of incurable disease, such as cancer or neurodegenerative diseases. However, the lack of effective drug target largely impedes the development of efficient therapies though target-therapy displayed success in certain cancer types. Despite the technologies and approaches have improved a lot, the bottleneck of target identification still existed in such an intricate biological system [21]. The costly and time-consuming target validation required elucidating the protein how to act on specific signaling pathway or process of disease [22]. Overall, identifying the direct target of the compound with advanced screening technique accelerated the process of new drug discovery. How to apply biological assays to novel drug research or how to search for new effective assays on target validation is no doubt a grand challenge in the field of drug devised.

### Application of HCS for drug discovery from traditional Chinese medicine

Over the past two decades, traditional Chinese medicine (TCM) has been considered to be a rich source of hit compound for drug development against the diversity of drug target validation. Hitherto, more than half of various drugs certified by FDA are derived from the natural source [23]. TCM and its derivatives had a profound effect on the treatment of the disease because of the diversity of biological activities [24].

Along with the improvement of high techniques, there were countless numbers of compounds have been isolated from TCM. Hence, it has become increasing difficult to identify completely new compounds with high bioactivity. Rediscovery is an unavoidable and necessary issue but it takes time and money [25]. Therefore, researchers were supposed to devise more efficient experimental assays and simultaneously enhance the screening technologies to identify the valued compound as a novel therapeutic agent. Besides the discovery, the isolation or purification of TCM was similarly a difficulty to defeat [26]. There are a myriad of components exist in TCM, how to isolate single compound, especially with low concentration, from complex compound libraries by chemical separation is still a conundrum [27]. Another intractable problem was how to purify the extract, and it also magnified the difficulties in drug discovery from TCM. In order to solve above issues, effective analytical method should be drawn up and improved to facilitate the drug discovery from TCM successfully.

Traditional Chinese medicine has historically become a rich source of discoverying novel drugs. Since high-content screening technology emerged as a robust tool in drug screening, the development of drug discovery from TCM has improved a lot. Efforts to search for therapeutic agents from TCM libraries have constantly continued in the past years. Researchers discovered some innovative drugs from large libraries with HCS approach to treat complex diseases. At present, more and more research institutions have applied HCS technology in the study of traditional Chinese medicines. HCS can not only clarify the interaction between the sieved samples and the drug targets, but also make it possible to understand other biological changes in the cell, predict the toxicity of the compound and investigate related metabolic pathways by observing the morphology of the cells, which are of great significance for promoting the modernization of traditional Chinese medicine. Recently, we have applied HCS technique in the discovery of autophagy regulator from traditional Chinese medicine for the therapeutic invention in the cancer and neurodegenerative disease models. Our studies demonstrate that the HCS technique is highly efficient and reliable for the identification of autophagy regulators. For example, Corynoxine B [28], Corynoxine [29] and curcumin analog C1 were identified as autophagy inducers while dauricine and daurisoline were identified as autophagy inhibitors [30].

There is an increasing number of studies applying HCS technique in the identification and pharmacological analysis of TCM-derived reagents. A comprehensive summarization of these studies is list in Table 2. Among the studies, the HCS has been used to identify therapeutic compounds for a wide range of human diseases including: cancer, neurodegenerative disease, neurotoxicity, osteoporosis, liver injury, liver fibrosis and inflammation. When analyzing the screening models, multiple cellular and molecular processes have been utilized in the screening as shown in Table 2. Several models mentioned in the table are extremely suitable for the HCS and we conclude here: (1) nuclear translocation of transcript factors: some transcript factors translocate to nuclear to initiate the gene transcription for down-stream cellular events thus the nuclear translocation of transcript factors can be used as a marker for transcription activation. Nuclear translocation of eIF4E, NFATc1, β-catenin, NF-κB and TEFB have been use as the readout for the identification of anti-cancer, anti-inflammation and neuroprotective compounds; (2) Morphological change of cells: Cell morphology changes are important marker of cell activation status, cell viability, cell growth and cell destiny. In the studies, the microtubules network, the outgrowth of neurites of neuronal cells and nuclei morphology have been monitored to evaluate the anti-tumor and neuroprotective properties of candidate compounds; (3) the function and distribution pattern of organelles: intracellular organelles play vital role in the cell survival and proliferation. The membrane potential of mitochondria and the distribution pattern of lysosomes have been used as markers for anti-cancer property evaluation of candidate compounds; (4) autophagosome formation: autophagy is a highly conserved cellular degradation process involved in neurodegenerative diseases and cancer. The formation of autophagosome has been widely used as marker for identification of autophagy regulators. In the listed studies, the autophagy inhibitors and inducers have been identified for the anti-cancer purpose and neuroprotective purpose. These successful examples strongly support the strength of HCS in the TCM research.

**Table 2:** Summary of HSC for drug discovery from TCM

Models	Principle	Platform	Purpose	Identified compound	Refs.
eIF4E immunostaining in MEF cells.	mTOR signaling pathway affects cellular localization of eIF4E	Array-Scan^®^ VTI HCS Reader (Thermo Scientific, USA)	Discover novel mTOR signaling pathway inhibitors	1,4-*O*-diferuloylsecoisola-riciresinol and PierreioneB	[31]
Nuclear translocation of NFATc1 in EGFP-NFATc1 U2OS cells	NFATc1 nuclear translocation affects osteoclast genesis and bone erosion	A confocal microscopy (Zeiss 710)	Screen for drug candidate for relevant diseases caused by osteoclast genesis	6-[10(Z)-heptadecenyl] salicylic acid from Syzygium tetragonum Wall	[32]
GFP-LC3-expressing HeLa cells	Autophagy induce the number of GFP-LC3 puncta rapidly increases	Opera HCS System, Columbus2.3 Software (Perkin-Elmer)	Screen for novel apoptosis and autophagy regulators	Neobractatin isobractatin from Garcinia bracteata	[33]
African green monkey kidney cell line BSC-1	Microtubules are essential for proper chromosome congression and separation	Automated microscope Axiovert 200 M (Carl Zeiss, Germany) data was analyzed using MetaMorph 7.7.8.0 (Visitron, Germany)	Search for inhibitor of mitosis	Podoverine A from the plant Podophyllum versipelle Hance	[34]
Primary cortical neuron and dorsal root ganglion	Morphological change of synapse and the neurite outgrowth reflect neuron viability	ImageXpress Micro automated wide-field fluorescent microscope (Molecular Devices)	Discover potential neuroprotective drugs for paclitaxel-induced neurotoxicity	5-Hydroxydecanoate (5-HD)	[35]
Cell cycle or cytoskeletal stain on HeLa cells	Cell cycle and cytoskeleton is the markers for cell growth and stress	ImageXpress Micro inflorescent microscope (Molecular Devices)	Develop new tool to identify novel compound from large candidate libraries	Quinocinnolinomycins	[36]
β-catenin staining in the human MG-63 OS cell line	β-catenin is the reporter of WNT signaling	(TFM-680; Shanghai Tuming Optical Instrument Co., Ltd.)	Identify WNT signaling inhibitor for the treatment of osteosarcoma	Resveratrol	[37]
The human prostate cancer cell line DU145	The position of lysosomes to the nucleus is a readout for lysosome anterograde trafficking	Cellomics Arrayscan (Thermo Fisher Scientific, Inc, Waltham, MA)	Screen for drugs that inhibit lysosome trafficking	Niclosamide	[38]
Non-small cell lung cancer (NSCLC) cell lines NCI-H157 and NCI-H460	Cell death can be monitored by Anexin V/PI and DAPI staining	Kinetic Scan Reader (ThermoFisher Scientific, USA)	Identify the compounds to induce cell death	Ophiopogonin B from Radix Ophiopogon Japonicus	[39]
p-ERK and c-myc staining in MKP-1 overexpression cells	p-ERK and c-myc reflect MKP-1 activity	ArrayScan II (Cellomics, Pittsburgh, PA) S-Plus statistical Software package (Insightful, Inc., Seattle, WA)	Identify the inhibitor of MKP-1	Sanguinarine	[40]
Apoptotic markers staining	Apoptotic markers reflect cells death	KineticScan Reader (Cellomics Inc, Pittsburg, PA, USA)	To identify hepatoprotective compounds	Salvianolic acid B from Radix Salviae miltiorrhizae	[41]
Neurite imaging of SH-SY5Y cells	Neurite outgrowth is a marker for ROCK2 inhibition	In Cell Analyzer 2000 (GE, Healthcare) IN Cell Investigator	Discover ROCK2 inhibitors	BIPM	[42]
LX-2 hepatic stellate cell proliferation	Hepatic stellate cell proliferation is a marker of liver fibrosis	Cellomics Arra yScan VTI HCS Reader Cellomics Cell Health Profiling BioApplication	Identify the active components of Danshen for anti-liver fibrosis	Salvianolic acid B, caffeic acid and rosmarinic acid	[43]
HepG2 cell	Apoptotic markers reflect cells death	Assay Scan VTI HCS Reader	Investigate the anti-proliferative and apoptotic effects of TMP	Tetramethylpyrazine	[44]
A549 cells	Nuclei morphology reflects cell growth and mitosis status	BeckmanCoulter/Q3DM EIDAQ100, Activity Base and XLfit software	Identify compound that induces mitotic arrest	A novel quinazolinone	[45]
Apoptotic markers staining on MCF7 cancer cells	Apoptotic markers reflect cells death	Eclipse TE2000-S microscope (Nikon, Tokyo, Japan)	Identify anti-cancer compounds	Koenimbin	[46]
The NF-κB translocation staining in HT-29 cells	Nuclear translocation of NF-κB reflects inflammation	Cellomic ArrayScan HCS Reader (Thermo Scientific, USA)	Determine the anti-cancer and anti-inflammatory effects of PAB	Pseudolaric acid B (PAB)	[47]
The NF-κB translocation staining in A549 lung cancer cells	Nuclear translocation of NF-κB reflects inflammation	ArrayScan HCS system (Cellomics), ArrayScan II Data Acquisition and Data Viewer version 3.0 (Cellomics)	Identify the NF-κB inhibitor	Panduratin A	[48]
Rhodamine 123 staining of mitochondrial membrane potential in H9c2 cardiac muscle Cells	Mitochondrial membrane permeability change affect Rhodamine 123 signal	Leica DMI 6000 B (Leica Microsystems, Wetzlar, Germany) Cell Detection 1.0 Software	Identify compound to rescue mitochondrial membrane potential decrease	Salvianolic acid B, protocatechuic aldehyde, and tanshinone II A	[49]
HeLa cells stably expressing EGFP-LC3	GFP-LC3 is the marker for Autophagosome	In Cell 2000 system (GE Healthcare)	Find therapeutic method in chemotherapy of cancer by inhibiting autophagy	Dauricine and daurisoline	[50]
Stable HeLa cells overexpressing 3×Flag-TFEB	TFEB nuclear translocation indicates biogenesis of autophagy-lysosome pathway	Opera high content system; Perkin-Elmer	Identify activators of TFEB	Curcumin derivative termed C1	[51]

## Discussion

In the past two decades, movements forward in instruments and software made the HCS become the powerful technique in drug discovery. HCS has in fact applied to identify drug candidates at various stages of the drug discovery pipeline: target validation, primary screening, candidate optimization and in vitro toxicology. It is undeniable that applying HCS to drug discovery from TCM facilitated the leading compounds identification, as well as the pharmacological study on the TCM. The application of the HCS elucidated the mechanisms, features and target points in individual cells or organisms and provided more insights into biological processes.

Although HCS technology improved a lot, scientists still have challenges on copious amounts of data analysis [52]. Thus, HCS dataset enable researchers to develop automated and advanced machine to quantify multiple cellular events or genetic information. When open source software gradually became more widespread, the analysis of HCS images seems to be more convenient and accurate. In addition, 3D tissue culture also has obstacles to surmount that 3D image processing software had difficulties in complicated 3D animal models. Along with the continuous progress of biotechnology, high-content screening will take a deeper optimization and become more authoritative and widespread. Though the robust assay was too overwhelming to handle a lot of barriers people met before, it is expected that future improvements in experimental approaches and computational instruments would add value to HCS in the future.

Traditional Chinese medicine contains thousands of compounds which regulate cellular function as a combination of multiple pharmacological activities, thus making it very challenging to understand the exact mechanism of drug activity. HCS provides a new technical means for studying Chinese medicine as a whole rather than as the isolated compounds, which better meet the complex factors of traditional Chinese medicine research and is in according with clinic use of TCM. Specifically, HCS has the following advantages for traditional Chinese medicine research: First, HCS can be used to study the function of cells as a whole, making the results more comprehensive and objective. Secondly, HCS can be used to screen multiple components of traditional Chinese medicine to find active components or components. Thirdly, HCS can help the exploration of the mechanism of traditional Chinese medicine from multiple levels and multiple targets, which are easily missed during traditional highly specific single-target screening. However, the difficulties of the application of HCS on TCM comparing to the pure chemical compounds are also obvious: (1) different compounds in TCM may compete with each other on the signaling pathways (inhibition or activation of same signaling pathway) or targets (agonist or antagonist) so as to mask the potential activity; (2) when a desired pharmacological activity were identified, it is difficult to figure out which compound is responsible that activity. Recent advance in the chemical biology and high-resolution separation science to fish the specific compound from the TCM pool use purified protein as bait is one of the solution for these difficulties. Moreover, the rapid development of “omics” approaches and pharmacological network analysis tool are making the attempt of deciphering the mystery of TCM much easier.

## Conclusion

HCS technology has been extensively invoked as a powerful tool for a rapid explosion in resolution and data processing both in the pharmaceutical industry or biological field. Traditional Chinese medicine has been valued as a rich source for drug discovery because of the well documented therapeutic efficacies since ancient time. However, lack of knowledge on the pharmacology mechanism and drug targets limited the further development of TCM. Applying the HCS technique will narrow the gap between therapeutic potential and molecular mechanism, thus strengthen the process for drug discovery from TCM.

## Abbreviations

TCM: traditional Chinese medicine
HCS: high content screening
HTS: high throughput screening
